# Perceptions of young men at the Free State School of Nursing with regards to teenage pregnancy

**DOI:** 10.4102/phcfm.v10i1.1358

**Published:** 2018-06-14

**Authors:** Siphiwe T. Madlala, Maureen N. Sibiya, Thembelihle S.P. Ngxongo

**Affiliations:** 1 Free State School of Nursing, Free State Province, South Africa; 2 Department of Nursing, Durban University of Technology, South Africa

## Abstract

**Background:**

Teenage pregnancy is a socio-economic challenge and a serious public health problem for communities in South Africa. It is, therefore, imperative that effective interventions and programmes be implemented to address this problem. A number of research studies have been conducted on teenage pregnancy in South Africa, but their focus was mainly on teenage girls excluding young men’s involvement in teenage pregnancy.

**Aim:**

The aim of the study was to determine the perceptions of young men between the ages of 18 and 23 years towards teenage pregnancy.

**Methods:**

A qualitative, explorative semi-structured interview descriptive design was used to conduct the study. The study was guided by the Johnson’s Behavioral System Model. Purposive sampling was used to select 10 participants with whom semi-structured interviews were conducted. Thematic analysis of data were performed.

**Setting:**

The study was conducted in Free State School of Nursing’s two main campuses.

**Results:**

The findings of this study revealed that young men were not involved in reproductive health programmes aiming to prevent teenage pregnancies. Father and son’s poor communication on issues related to sex and teenage pregnancy contributes to unprotected sexual intercourse resulting in teenage pregnancy. Cultural and traditional practices such as the withdrawal method, not using contraceptives, and misleading teachings at the initiation schools contribute to risk factors of teenage pregnancy.

**Conclusion:**

There is still a gap in reproductive health that needs to be filled by involving young men to reduce teenage pregnancies. Involving young men in reproductive health programmes could lead to a decreased number of teenage pregnancies. Factors, such as cultural and traditional practices, and father and son sexual health education, need to be taken into consideration to prevent teenage pregnancies.

## Introduction and background information

Teenage pregnancy rates in South Africa remain high, as around 30% of 15–19-year olds have reportedly ever been pregnant.^1^ The household survey statistics conducted in South Africa in 2010 revealed that approximately 32.5% of girls aged 15–19 years were or had been pregnant at least once during 2011.^2^ In 2011 and 2012, respectively, 31.5% and 31.4% girls were reported to be pregnant in South Africa.^2^ This high incidence of teenage pregnancies indicates that there might be a greater measure of sexual freedom among South African teenagers. Reducing the incidence of teenage pregnancy is an important focus of the agenda of action for meeting most of the Millennium Development Goals (MDGs), designed to halve extreme poverty by 2015.^1^

The National Department of Education (NDE) embarked on a Sexual Education Programme (SEP) that formed part of the Life Orientation learning area during 2002.^3^ Learners are taught about sex-related issues at schools through Life Orientation as a subject.^3^ Despite being taught about sexual education at schools, teenage pregnancy still remains problematic in South Africa.^4^ When dealing with teenage pregnancy, the attention is often focused solely on girls, and young men are being disregarded although they play a critical role in teenage pregnancies.^4^ Young men are the initiators of sexual intercourse and influence the relationship in terms of having sex.^4^

Because of the behavioural underpinnings of teenage pregnancy, the Johnson’s^5^ Behavioral System Model was chosen to guide the study. This model was selected based on the notion that nursing is an external regulatory force that acts to preserve the organisation and integration of the patient’s behaviour at an optimal level under those conditions in which the behaviour constitutes a threat to physical or social health, or in which illness occurs.^5^ Human behaviour consists of emotional, physiological and physical behaviours that are categorised into seven subsystems,^5^ which are as follows:

- Attachment or affiliation: This is regarded as the first response of an individual’s system. It allows social inclusion, intimacy, formation and maintenance of a strong bond and provides an individual with a sense of security.
- Dependency: This involves an individual’s attainment of approval, attention, recognition or physical assistance from others.
- Ingestive: This involves the biological behaviour surrounding the intake of food and what is socially acceptable in a given culture.
- Elimination: This involves the physical behaviour surrounding the excretion of waste products from the body.
- Sexual: This involves psychological behaviour related to procreation, attraction and fulfilment of expectations associated with one’s sexuality, caring for others and to be cared for by others.
- Aggression: This involves the emotional behaviour concerned with protection and self-preservation that generate defence responses when an individual’s life or territory is threatened.
- Achievement: It provokes behaviours that attempt to control the environment.

In this study, attachment, achievement, dependency, aggression and sexual system were used to explore and describe the factors that play a critical role in teenage pregnancy which are in line with Johnson’s Behavioral System Model. These factors are as follows:

- poor communication, especially father-to-son communication
- misinterpretation of cultural and traditional sexual practices
- involvement of young men in reproductive health and sex education.

### Problem statement

Teenage pregnancy at schools remains a problem across South Africa.^2^ Traditionally, pregnancy prevention and reproductive health services have been female-focused, and young men have been excluded in these programmes.^6^ However, since the onset of the Acquired Immune Deficiency Syndrome (AIDS) pandemic, there has been a greater need for young men between the ages of 18 and 23 years, to be involved in teenage pregnancy prevention programmes and in reproductive health issues. Young men believe that they are physically ready to engage in sexual intercourse but being physically ready does not always mean that they have enough knowledge about sexual activities and the consequences thereof.^7^

Sex is a topic that is not often addressed by family members with their teenagers, especially not with young men.^8^ This could lead to them engaging in risky sexual behaviours and could result in contracting sexually transmitted infections (STIs) and teenage pregnancies.^8^ Studies revealed that 38% of parents thought that talking about sexuality would encourage sexual acts among teenagers.^8^ Involvement of young men in the sexual education programmes and effective communication about sex could help to implement positive interventions resulting in reduced numbers of teenage pregnancies.^3^

### Aim of the study

The aim of the study was to determine the perceptions of young men between the ages of 18 and 23 years at the Free State School of Nursing with regard to teenage pregnancy.

### Objectives of the study

The objectives of the study were to: identify the roles played by young men in teenage pregnancy, the risk factors influencing young men’s perceptions, and preventative practices regarding teenage pregnancy.

## Research method

### Study design

A qualitative research design was used to explore and describe the perceptions of young men regarding teenage pregnancy.

### Study setting

The Free State School of Nursing consists of the Eastern, Northern and Southern campuses situated within the Free State Province. The study was conducted at two main campuses of the school, which are the Northern and the Southern Free State School of Nursing.

### Sample and sampling technique

The two main campuses namely, Northern and Southern Free State School of Nursing, were used to select the participants. The Eastern Free State School of Nursing campus participants formed part of the pilot study, therefore, they were excluded from the main study population. This was followed by purposive sampling of a total of 10 young men enrolled in the Diploma in General Nursing (Community, Psychiatry) and Midwifery course at both campuses. The young men; who met the inclusion criteria and agreed to participate in the study, were included.

### Inclusion and exclusion criteria

The study included young men who were between the ages of 18 and 23 years, currently registered as student nurses at Free State School of Nursing studying towards a four-year comprehensive Diploma in Nursing. Female students and all young male students below the age of 18 years and above 23 years of age studying at the Free State School of Nursing were excluded from the study.

### Pretesting of the interview guide

A pretest was conducted at the Eastern Campus Free State School of Nursing in order to determine the feasibility of the study and the relevance of the interview guide. Two participants, who met the inclusion criteria, were purposefully selected to be included in the pretesting of the interview guide. A semi-structured interview was conducted with the first participant, and the results were confirmed by conducting a semi-structured interview with a second participant. The pretest participants understood the questions, although the researcher had to probe to obtain more information from their responses. Data were analysed and interpreted. No changes were made to the study processes and the interview guide. The pretest site, the participants and the results, were not included in the main study.

## Data collection

Semi-structured interviews with young men; were conducted at Free State School of Nursing in a private room at each campus. Data were collected from the beginning of November 2014 - December 2014. Data collection focused on the following themes and were guided by Johnson’s Behavioral System Model:

- Demographic data such as; participants’ age, race, marital status and number of own biological children were collected in order to determine the dependency to the environment.
- Participants’ perceptions regarding teenage pregnancy to determine their perceptions regarding sexual activities and their roles in teenage pregnancies.
- Participants’ opinions regarding the following risk factors were explored and described:
  ▪ alcohol, smoking cigarettes, marijuana and the use of other drugs
  ▪ cultural and traditional beliefs about sexual practices
  ▪ socio-economic status
  ▪ knowledge and use of contraceptives
  ▪ sex education taught by parents at home and teachers at schools.
- This was performed to determine young men’s behaviours such as: aggression, attachment, achievement and dependency caused by these factors that lead to teenage pregnancies.
- Participants’ culture, traditional practices and beliefs that lead to misconceptions and misinterpretations about sex and teenage pregnancies.
- Participants’ comments and suggestions regarding teenage pregnancies, in order to identify measures to prevent teenage pregnancy.

The Lincoln and Guba’s^9^ Model was used to ensure trustworthiness. Credibility was ensured by investing more time in the field and prolonging the length of engagement with the participants.^10^ The researcher returned to the selected Free State School of Nursing campuses to ask the participants to review the analysis and interpretation of data to ensure member checking. Young men’s own truthful verbatim accounts regarding their perceptions on teenage pregnancy were captured and analysed, and the findings were subjected to an audit trial to ensure dependability. The relevance of data was audited by an external auditor not affiliated to the university to eliminate potential subjectivity and bias of the researcher to ensure confirmability. Description of the research setting, sufficient data from 10 young men and research processes used were provided to ensure transferability of the study findings.

### Data analysis

Data were analysed thematically.^10^ Themes and subthemes that emerged from the informants’ perceptions regarding teenage pregnancies were pieced together to form a comprehensive picture of their collective experiences.

## Ethical considerations

Approval for the study was obtained from the ethics committees of the institutions (REC 53/14). Letter of information was issued to all the participants, and all the participants who agreed to participate in the study had to sign an informed consent form. Permission to record the interviews was obtained from all the participants before data collection commenced.

## Research study findings

After analysed data, the findings revealed several themes and subthemes that emerged as evident from Table 1.

**TABLE 1:** Themes and subthemes.

Themes	Subthemes
Theme 1: Perceptions regarding teenage pregnancies	Home upbringing and support Roles played by young men in teenage pregnancies
Theme 2: Risk factors for teenage pregnancies	Abuse of alcohol and drugs Poor socio-economic status
Theme 3: Cultural and traditional practices	Misinterpretations concerning circumcision Cultural beliefs and misconceptions about sexual intercourse

### Theme 1: Perceptions regarding teenage pregnancies

The findings under this theme were varied but pointed on the issues pertaining how they were brought up at home and the support they receive from their parents as young men regarding sex-related issues.

#### Home upbringing and support

Most of young men were of opinion that the type of family structure and support they receive at home and from the community were imperative in their upbringing. However, there was lack of father-to-son communication when coming to issues related to sexual education. One participant noted:

‘… my father never discussed any issues related to sex topic with me’ (Participant 4, 20 years old, black person).

Their expressions were that there was a lack of communication on issues related to availability and use of contraceptives including discussion about sex topics, as they were regarded as taboo in most family dwellings:

‘Sex education or talks with parents does not exist especially when you are raised by a grandmother. Sometimes you need some sort of support and guidance on issues related to sex but you don’t know who to turn to.’ (Participant 1, 22 years old, black person)

#### Roles played by young men in teenage pregnancies

Young men reported that when coming to reproductive health, they felt left out as this was still regarded as a female domain. They strongly believed that they also played a crucial role in teenage pregnancies as they took part in conception of the baby. They expressed their views by stating that:

‘I have an active role to play in teenage pregnancy prevention as an initiator of the relationship. When I date a girl, if we agreed to wait until we are married before we engage in sexual intercourse, it is my role to stick to the agreement and respect her not to pressurise her to have sex with me.’ (Participant 6, 21 years old, black person)

Other young men believed that they experience pressure from their friends to engage in sexual intercourse and that could lead to teenage pregnancy or contracting sexually transmitted diseases including Human Immunodeficiency Virus (HIV). This was captured in this response:

‘In most case we are being pressurised by our friends to engage in an unprotected sex with our girlfriends putting ourselves at risk of impregnating them and sometimes contracting sexually transmitted diseases including being HIV positive. But it is our role and responsibility to refuse to do things to please our friends.’ (Participant 2, 22 years old, mixed race person)

### Theme 2: Risk factors for teenage pregnancies

Several risk factors were raised by young men that might influence them to engage in unprotected sexual intercourse; exposing them to risks of contracting sexually transmitted diseases and causing unplanned teenage pregnancies.

#### Abuse of alcohol and drugs

Most young men indicated that the use of alcohol and drugs were the main contributing factors for engaging in unprotected sex leading to teenage pregnancies. They alluded that when one is under the influence of alcohol and drugs, one’s mind becomes clouded. This impacts negatively one’s rational decision-making capabilities. Therefore, chances of using protection at this point are limited. This was expressed in the following excerpt:

‘When you are drunk or used drugs, you do not think rationally. You end up picking any other drunken girl in the tavern and have sexual intercourse with her without using the condom, the next thing she is pregnant, and you may also be infected with sexually transmitted diseases.’ (Participant 8, 20 years old, black person)

#### Poor socio-economic status

The participants indicated that they were brought up in various life challenges, which includes, among others, poor socio-economic environments. These circumstances had an impact on their upbringing including their sexual practices. Some indicated that their home conditions affected them psychologically as they induced stress to them. Hence, engaging in sexual activities was their escape in dealing with stressful situations at home. Participants expressed this:

‘Growing up in a poverty-stricken home is very difficult. I was raised by a single parent mother and financially we were struggling. This put so much pressure and stress on me. Therefore, to relive this sometimes I ended up engaging in sexual intercourse to distress.’ (Participant 9, 22 years old, black person)

Fathering a child was seen as another way of financial freedom to young men as they will share the child social grant money with their girlfriends to support them at home. One participant narrated the following:

‘… some of my friends stated that they make kids with their girlfriends to share the child grant to support their parents.’ (Participant 10, 21 years old, black person)

### Theme 3: Cultural and traditional practices

Participants were concerned about cultural and traditional practices they undergo at the initiation schools which groom them to become men.

#### Misinterpretations concerning circumcision

Young men indicated; that some of the teachings they received at the initiation schools were misleading. These teachings were mostly given by elders who believed that talking about sex-related topics were taboo. The meaning of these teachings was believed to be leaving young men with many unanswered questions; which they were afraid to ask from the elders at the initiation schools.

This was captured in this verbatim:

‘Do not have sexual intercourse immediately after leaving the initiation school, but you are a man now. They do not tell you when to resume sexual intercourse, how to use protection and if the sexual intercourse will still be the same as before circumcision. Hence, most boys engage in unprotected sexual intercourse to validate this.’ (Participant 1, 22 years old, black person)

Although other participants narrated that after being circumcised by the elders at the initiation schools, they had preconceived ideas that they would be taught about sex and the use of contraceptives; including how to prevent teenage pregnancies and sexually transmitted diseases. This was the expectation they had about the initiation schools which could have filled the gap left by their parents regarding these issues:

‘I was expecting to be taught about various issues concerning sexual intercourse, how to protect myself from sexually transmitted diseases including avoiding teenage pregnancy since my mother never talked to me about such issues.’ (Participant 1, 22 years old, black person)

#### Cultural beliefs and misconceptions about sexual intercourse

Most cultures believe that pregnancy can be prevented by abstaining from engaging in sexual activities. Young men agreed to this notion, but they were concerned that their parents do not engage with them about such teachings. Mostly sexual abstinence was emphasised to girls, that they should preserve their virginity until they get married. This was what the participant had to say:

‘… sexual abstinence is only discussed and encouraged to be practiced by girls. They are taught to preserve their virginity until they get married. Whereas with boys our parents do not talk about how we should preserve our virginity and the benefits thereof. Hence boys tend to compete with one another to have unprotected sex with virgins as this practice is perceived as an achievement, but this could lead to teenage pregnancy and contraction of sexually transmitted diseases.’ (Participant 7, 20 years old, black person)

Some of the participants mentioned, that there were several cultural practices that could lead to misconceptions about sexual intercourse. Cultural sexual practices; such as the withdrawal method, could pose a threat to young men’s lives in believing that practicing the withdrawal method during sexual intercourse will prevent teenage pregnancy. The misconception of this cultural sexual practices influences young men to indulge in unprotected sexual intercourse. This practice does not only pose the danger of impregnating girls but also expose both young men and their partners to the risk of contracting sexually transmitted diseases including HIV. This was illustrated by the following excerpt:

‘It is believed by many boys that if you engage in sexual intercourse and practise withdrawal method you will not cause unplanned teenage pregnancy. This is very risky as many boys believe in and practice this unreliable cultural sex method which can lead to teenage pregnancy including contraction of sexually transmitted diseases.’ (Participant 2, 22 years old, mixed race person)

## Discussion

The finding of the study revealed that there were various factors that contributed to young men engaging in unprotected sexual intercourse. These factors range from young men’s home upbringing and support received from parents, roles played by young men in teenage pregnancy prevention, use of alcohol and drugs, socio-economic status including cultural and traditional practices. These findings are discussed as follows:

### Perceptions regarding teenage pregnancies

It became evident from the study’s findings, that the home environment plays an important role in the upbringing of the child as it gives a state of dependency. According to Johnson’s^5^ Behavioral System Model, state of dependency is regarded as a state of relying on or needing someone from the environment for aid, support, reliance, confidence or trust. Most of the participants were brought up by their single mothers or by their grandmothers in the absence of their fathers. Therefore, they were deprived of an opportunity to be supported, aided and guided by their fathers on issues related to sexual health. It was attested that men who are deprived of a father figure during their early lives are likely to engage later in rigidly overcompensated masculine behaviour, including engaging in unprotected sexual practices.^11^ The study findings also revealed that despite the presence of a father figure in their upbringing, there was a lack of communication between father and son on issues related to sex education. Parents were seen to be sceptical to discuss sexual issues with their children as this was regarded as taboo. Parents not only find it taboo to discuss sex-related topics with their children but, they also perceive sex as an embarrassing topic to discuss.^12^ Hence, young men rely on their peers, media and magazines as a source of information. The information from these sources could be misleading and can contribute to unprotected sexual intercourse, resulting in unplanned teenage pregnancies.

Young men believed that they have a crucial role to play in the prevention of teenage pregnancy if they were actively involved in reproductive health issues. Traditionally, family planning and reproductive health services have been female-focused.^6^ However, the study findings argued that young men need to actively partake in reproductive health to change this traditional mindset. Studies revealed that; by not working with men, the health care system misses more than half the equation, as men are influential in the sex-related activities that put them at risk of causing teenage pregnancies, including contracting sexually transmitted diseases and HIV.^13^

### Risk factors for teenage pregnancies

Young men perceived alcohol and drug use as the most contributing factors to teenage pregnancies. They narrated that these substances have an impact on the physiological level of functioning. The findings revealed that the intoxication effects caused by these substances affect the thinking level of the user; exposing one to the risk of engaging in unprotected sexual intercourse with random girls picked from the clubs. It was cautioned that the psychoactive effects of alcohol and drugs are thought to increase sexual arousal and desire, decrease inhibition and tenseness, diminish decision-making capacity, judgement and sense of responsibility.^14^ This increases the chances of engaging in risky sexual practices leading to unplanned teenage pregnancy including contracting sexually transmitted diseases and HIV.

Among other risk factors identified from the study findings, poor socio-economic status was one of the main points of concern. The findings indicated that most participants’ home environments which they grew up in were very stressful because of poverty. In the study conducted in the Capricorn District of the Limpopo province, approximately 44% of the households depended on single mothers’ income, 16% depended on child support grants and 8% were living on their grandparents’ pension grants.^15^ These sources of income were inadequate to meet all the needs of the families. This could be a stressful situation; hence, the study findings revealed that most of young men use sexual intercourse as a stress relieving method that can lead to teenage pregnancies and the risk of contracting sexually transmitted diseases. Other authors support this notion by indicating that lack of necessary resources to meet the needs of the household places adolescence at greater risks of teenage pregnancy.^16^

Young men are faced with different life challenges as they grow up, including peer pressure. Peer pressure plays an important role in their social development as it provides them with the sense of belonging and security. This is a form of attachment as described by Johnson’s^5^ Behavioral System Model that attachment is the first response system to develop in an individual. This allows inclusion, intimacy, formation and maintenance of strong bonds as well as providing an individual with a sense of security. Therefore, the study findings revealed that fathering a child was seen as a source of income as encouraged by peers, with the notion that young men and their girlfriends could share the child social grant to support their respective home dwellings to alleviate poverty. This incorrect information about sex from the peer group, peer pressure or the need of belonging and security to fit with peers, increases the rate of unwanted teenage pregnancies.^17^

### Cultural and traditional practices

Culture plays a significant role in perpetuating the values and norms of a society, yet also offers important opportunities for creativity and change.^18^ Some of these values and norms are culturally believed to be taught to young men by traditional elders during their initiation at the mountains. However, it is not clear which values and norms are taught to young men at the initiation school as what happened at this cultural practice is kept a secret. Although the study findings revealed that elders at the initiation school do not dwell much on issues pertaining to sex and teenage pregnancy prevention, it became evident from the participants’ responses that they were left with many unanswered questions after circumcision. Powerful taboos are attached to the discussion of circumcision rites with outsiders, and this has meant that until relatively recently, the subject has not been widely researched or discussed in South Africa.^19^ It was further revealed from the study findings that traditional elders do not talk about sex and how to prevent teenage pregnancy except telling the initiates that ‘… now you are a man’. After circumcision, the education given to young men was limited only to personal hygiene, self-control and general social morals.^20^ Hence, young men resort in engaging in unprotected sexual intercourse to ascertain if their manhood and if sexual intercourse was still the same as before circumcision. By doing so; they are exposing themselves to the risks of contracting sexually transmitted diseases including causing unplanned teenage pregnancy.

Traditionally, abstinence is a worldwide practice whereby elders encourage teenagers to preserve their virginity until they get married. Although this practice is encouraged to be practised worldwide, it is more emphasised to girls than boys. The findings of this study attested to this notion that abstinence to sexual intercourse was more focused on girls than young men. Abstinence was the major cause of declining birth and teenage pregnancy among girls; hence, this decline in teenage pregnancy could increase if abstinence was also filtrated to young men.^21^ Besides abstinence, study findings raised concerns about the cultural sexual withdrawal method that has been practised over decades and believed to be effective in preventing pregnancies. This method is believed to be extremely risky, as young men including their partners could contract sexually transmitted diseases and HIV and most likely cause teenage pregnancy.

### Limitations of the study

The study was conducted at two main campuses of the Free State School of Nursing situated in the Free State Province; therefore, these findings are not transferable to other provinces or to other schools of nursing. The interviews were conducted only with the young men between the ages of 18 and 23 years enrolled for a 4-year comprehensive diploma course in nursing (R425). Other students were not included in the sample, although young men who are not student nurses might have different perceptions about teenage pregnancies.

### Recommendations

Young men need to be actively involved in reproductive health. There should be open communication channels with their parents especially their fathers or any male figure that could impart knowledge regarding sex and its consequences. Single parents raising boys should form support groups to deal with issues that pertain to young men so that they can be able to give sexuality education to their sons without feeling embarrassed or believing that it is taboo. There should be collaboration between health care workers, elders at the initiation schools, parents and school teachers to facilitate sexuality education for all children including young men. Policies developed by the Department of Health regarding reproductive health need to be revised to include men’s involvement in reproductive health and family planning issues. Traditional initiation schools need to be regulated by the Department of Health in order to guide the initiators towards giving guidance to young men about safe sexual practices after initiation.

## Conclusion

It was clear from this study that there is still a gap in reproductive health that needs to be filled by involving young men to reduce teenage pregnancies. Young men play active roles in teenage pregnancies; therefore, it is important that they are included in programmes concerning reproductive health care. This will assist them to gain more knowledge regarding sex-related issues so that they can be able to take sound decisions before engaging in unprotected sexual activities. The findings of this study make a valuable contribution to reproductive health care by making an effort to fill the gaps in existing literature on teenage pregnancy.
